# Inhibition of p70 Ribosomal S6 Kinase (S6K1) Reduces Cortical Blood Flow in a Rat Model of Autism-Tuberous Sclerosis

**DOI:** 10.1007/s12017-024-08780-7

**Published:** 2024-04-04

**Authors:** Oak Z. Chi, Xia Liu, Harvey Fortus, Guy Werlen, Estela Jacinto, Harvey R. Weiss

**Affiliations:** 1 Department of Anesthesiology and Perioperative Medicine, Rutgers Robert Wood Johnson Medical School, 125 Paterson Street, Suite 3100, New Brunswick, NJ 08901-1977 USA; 2 Department of Biochemistry and Molecular Biology, Rutgers Robert Wood Johnson Medical School, Piscataway, NJ 08854 USA; 3 Department of Neuroscience and Cell Biology, Rutgers Robert Wood Johnson Medical School, Piscataway, NJ 08854 USA

**Keywords:** p70 ribosomal S6 kinase, Cerebral blood flow, Tuberous sclerosis complex, Autism spectrum disorders, PF-4708671

## Abstract

The manifestations of tuberous sclerosis complex (TSC) in humans include epilepsy, autism spectrum disorders (ASD) and intellectual disability. Previous studies suggested the linkage of TSC to altered cerebral blood flow and metabolic dysfunction. We previously reported a significant elevation in cerebral blood flow in an animal model of TSC and autism of young Eker rats. Inhibition of the mammalian target of rapamycin (mTOR) by rapamycin could restore normal oxygen consumption and cerebral blood flow. In this study, we investigated whether inhibiting a component of the mTOR signaling pathway, p70 ribosomal S6 kinase (S6K1), would yield comparable effects. Control Long Evans and Eker rats were divided into vehicle and PF-4708671 (S6K1 inhibitor, 75 mg/kg for 1 h) treated groups. Cerebral regional blood flow (^14^C-iodoantipyrine) was determined in isoflurane anesthetized rats. We found significantly increased basal cortical (+ 32%) and hippocampal (+ 15%) blood flow in the Eker rats. PF-4708671 significantly lowered regional blood flow in the cortex and hippocampus of the Eker rats. PF-4708671 did not significantly lower blood flow in these regions in the control Long Evans rats. Phosphorylation of S6-Ser240/244 and Akt-Ser473 was moderately decreased in Eker rats but only the latter reached statistical significance upon PF-4708671 treatment. Our findings suggest that moderate inhibition of S6K1 with PF-4708671 helps to restore normal cortical blood flow in Eker rats and that this information might have therapeutic potential in tuberous sclerosis complex and autism.

## Introduction

Tuberous sclerosis complex (TSC) is a genetic disorder marked by the formation of multiple benign tumors arising from mutations in *TSC1* or *TSC2* genes. Epilepsy is the most common sign of human TSC (about 90%) followed by autism spectrum disorders (ASD) (50%) and intellectual disability (45%) (Curatolo et al., 2023; Henske et al., 2016; Peters et al., 2013). TSC protein complexes that include *TSC1* and *TSC2* function as inhibitors of the mammalian target of rapamycin (mTOR) signaling pathway (Curatolo et al., 2022; Huang & Manning, 2008). Defect in *TSC1* or *TSC2* proteins induces overactivation of mTORC1 (Curatolo et al., 2022; Henske et al., 2016; Mizuguchi et al., 2021). mTOR is a component of two protein complexes, mTORC1 and mTORC2, playing a crucial role in regulating cell growth and metabolism. Of the two complexes, mTORC1, comprised of mTOR, raptor, and mLST8, is more comprehensively understood, primarily due to the inhibitory impact of rapamycin on this complex. One of the most extensively studied targets of rapamycin-sensitive mTORC1 is the p70 ribosomal S6 kinase (S6K1), a protein kinase that phosphorylates the ribosomal subunit protein S6, which regulates translation initiation. Rapamycin-mediated inhibition of mTORC1 leads to the downregulation of S6K1 phosphorylation and activation. In contrast, mTORC2, which is not directly affected by rapamycin, responds to growth factors and facilitates full activation of Akt through phosphorylation (Oh & Jacinto, 2011). Extended rapamycin exposure indirectly hampers mTORC2 and Akt phosphorylation by obstructing the assembly of newly synthesized mTOR with its mTORC2 components, including rictor, SIN1, and mLST8 (Sarbassov et al., 2006).

Since increased activation of mTORC1 could contribute to the manifestations of TSC such as epilepsy, intellectual disability and ASD, pharmacological inhibition of mTORC1 may be effective in preventing and reducing TSC associated neuropathological symptoms (Mizuguchi et al., 2021; Winden et al., 2018). Rapamycin and its second generation everolimus have been shown to reduce seizure susceptibility and autism-like behavior in several animal models of TSC (de Vries, 2010; Ehninger et al., 2008; Koike-Kumagai et al., 2022; Talos et al., 2012; Tsai et al., 2012; Zeng et al., 2008). Rapamycin has been tested in humans with ASD, with some potential benefit (Cavalheiro et al., 2021; Franz et al., 2021; French et al., 2016; Krueger et al., 2018; Mizuguchi et al., 2021; Winden et al., 2018). However, there are significant side effects with rapamycin which limit its use (Benjamin et al., 2011; Ganesh & Subathra Devi, 2023; Li et al., 2014; Magaway et al., 2019). We have chosen to examine inhibition of a specific target of mTORC1, that is S6K1, to see if there is any effect on cerebral blood flow in a rodent model of TSC, Eker rats.

The Eker rat, carrying a spontaneous germline mutation in *TSC2* gene (*Tsc2* ±) (Curatolo et al., 2022, 2023; Henske et al., 2016; Peters et al., 2019), has been an invaluable model for understanding TSC (Habib, 2010; Kútna et al., 2020; Wenzel et al., 2004). Research has demonstrated that this animal model exhibits comparable molecular defects observed in other mouse models of TSC and ASD. Additionally, autism-like behavioral abnormalities have been noted in the Eker rats (Waltereit et al., 2011).

We have previously demonstrated that young Eker rats exhibit significantly increased basal cerebral oxygen consumption and blood flow (Weiss et al., 2007). Our preliminary investigations also indicated that the heightened cerebral metabolism and blood flow in the young Eker rats were not affected significantly by *N*-methyl-D-aspartate (NMDA) or α-amino-3-hydroxy-5-methyl-4-isoxazolepropionic acid (AMPA) receptor blockade or stimulation (Weiss et al., 2007, 2009, 2012). In contrast rapamycin significantly reduced the basal elevated cerebral blood flow and metabolism in the Eker rat to the level of the control rats (Chi et al., 2015). Based on our earlier data, it appears that modifying the excitation/inhibition balance in synapses did not significantly contribute to the increased regional cerebral blood flow (rCBF) in Eker rats. Instead, the heightened rCBF in Eker rats can be attributed to the overactivity of the mTOR pathway. There is a possibility that blocking a key target component of mTORC1 signaling, S6K1, could yield similar effects.

The aim of this study was to investigate whether inhibiting the activity of S6K1 with PF-4708671 in Eker rats could decrease the elevated regional cortical blood flow associated with this autism-tuberous sclerosis model. We assessed regional cerebral blood flow in Long Evans (control) and Eker rats and examined components of the mTOR signaling pathways. PF-4708671 did not cause a significant reduction in cortical blood flow in the control Long Evans rats. As our model of tuberous sclerosis-ASD, the Eker rats exhibited significantly higher cortical blood flow compared to Long Evans rats. Treatment with PF-4708671 significantly lowered the blood flow rate in the Eker rats, suggesting a correlation with reduced mTOR signaling.

## Materials and Methods

### Animals

As described previously (Chi et al., 2015; Weiss et al., 2007, 2009), this study was conducted following the guidelines set forth by the US Public Health Service, as outlined in the Guide for the Care of Laboratory Animals (DHHS Publication No. 85-23, revised 1996), and received approval from our Institutional Animal Care and Use Committee. Sixteen male Long Evans rats (90–100 g) from Charles River Laboratories (Wilmington, MA) and 16 male Eker rats (90–120 g) from Center for Precision Environmental Health, Baylor College of Medicine (Houston, TX) were randomly divided into vehicle-treated (n = 8) and PF-4708671 (S6K1 inhibitor) treated (n = 8) groups for the cerebral blood flow segment of the study. Long Evans rats were utilized as the control group for this experiment. These animals were approximately 5 weeks old and were used to measure cerebral blood flow. Additionally, six Long Evans and six Eker rats were employed for biochemical analysis in this study.

### Animal Preparation

As we described previously (Chi et al., 2015), the rats were anesthetized with a 2% isoflurane-air-oxygen mixture via tracheal tube and mechanical ventilation to maintain arterial PO_2_ above 100 mm Hg and PCO_2_ around 35 mmHg. A femoral artery and a femoral vein cannulation were performed. The venous catheter was utilized for administering the radioactive tracer, while the arterial catheter was connected to a Statham P23Db pressure transducer and an Iworx data acquisition system to monitor heart rate and blood pressure. Arterial blood samples were collected through this catheter for analysis of hemoglobin, blood gases, and *p*H using a Radiometer blood gas analyzer. Body temperature was maintained at 38 °C using a servo-controlled rectal thermometer probe and a heating lamp. After the surgery, isoflurane concentration was reduced to 1.4%.

In the PF-4708671 treated animals, 75 mg/kg of PF-4708671 (Sigma-Aldrich) dissolved in 20% DMSO plus 10% Tween-80 in normal saline (vehicle) was injected intraperitoneally one hour before cerebral blood flow measurements. In the control group, the vehicle was injected. Just before determination of regional cerebral blood flow, the final arterial blood pressure and heart rate were recorded, and an arterial blood sample was drawn anaerobically for analysis of PO_2_, PCO_2_, *p*H, and hemoglobin concentration.

### Regional Cerebral Blood Flow

As we previously described (Chi et al., 2015; Weiss et al., 2007, 2009), for the determination of cerebral blood flow, 20 μCi of ^14^C-iodoantipyrine was intravenously infused through the femoral venous catheter. As the isotope entered the venous circulation, the femoral arterial catheter was trimmed to about 20 mm in length. Arterial blood samples of 10 µl each were collected into the microtubes from the femoral arterial catheter approximately every 3 s for 60 s. Immediately after the final sample was collected, the rats were decapitated, and their heads were rapidly frozen in liquid nitrogen. The brains were dissected into four regions: cortex, hippocampus, cerebellum, and pons. Brain samples were sliced into 20 μm sections using a microtome-cryostat in −20 °C and dried immediately before exposure to X-ray film for 4 days to produce an autoradiogram. The ^14^C-iodoantipyrine concentrations in the tissues were determined by referencing eight precalibrated standards (40–1069 nCi/g, Amersham) through a computer-based microdensitometer system. Each brain region was subjected to at least eight average density measurements. Blood samples were solubilized in tissue solubilizer and, after 24 h, transferred to a counting fluid. The isotope counts were quench-corrected. Regional blood flow calculations were based on the following equation:

$$Ci\left(T\right)=\lambda K\int {{C}_{A}{e}^{-K(T-t)}dt;}$$

where *Ci*(*T*) represents the tissue concentration of ^14^C-iodoantipyrine at the time of decapitation, *λ* is the tissue:blood partition coefficient, *C*_*A*_ is the arterial concentration of the tracer, *t* denotes the time, and *K* is defined as *K* = *mF*/*λW*, where *m* is a constant, *F*/*W* represents the flow per unit mass of tissue, and a *λ* value of 0.80 was utilized.

### Western Blot

To analyze total protein extracts, brain tissue was lysed in RIPA buffer (100 mM NaCl, 50 mM Tris pH 8, 1% TX-100, 0.2% SDS, 0.5% sodium deoxycholate, 5 mM EDTA, supplemented with phosphatase and protease inhibitor cocktail). The lysates were centrifuged at 16,000 g for 10 min at 4 °C. Protein concentration was determined by Bradford analysis. About 30 µg of extracts from each sample were resolved by SDS-PAGE followed by transfer to PVDF membranes and immunoblotting using the indicated antibodies. All antibodies were used at 1:1000 dilution except for β-actin antibody (1:5000). Antibodies to pS240/244-S6 (5364S), S6 (2317S) Akt (9272S), pS473-Akt (4060S), were from Cell Signaling (Danvers, MA); β-actin (sc-47778) was from Sta. Cruz Antibodies (Santa Cruz, CA). Immunoblots were processed using Dura Supersignal enhanced chemiluminescence horseradish peroxidase substrate (Fisher PI-34076). Images were captured using Amersham Imager 600. ImageJ software [NIH; version 1.51 (100)] was used for densitometric analysis of protein expression or phosphorylation. The amount of phosphorylation was normalized to corresponding total protein levels. Results (in arbitrary units) were plotted, and statistical analysis conducted using GraphPad Prism 9 using two-way ANOVA followed by Tukey’s post-hoc test.

### Statistics

As we described previously (Chi et al., 2015), a one-way analysis of variance was employed to assess the differences between treatment groups in the Eker and Long Evans rats for various hemodynamic and blood gas parameters. A two-way analysis of variance was utilized to analyze blood flow between the groups and among multiple brain regions within the group. Post hoc multiple comparisons were conducted using Tukey’s procedure. All values are presented as mean ± S.E.M. A significance level of *p* < 0.05 was considered statistically significant.

## Results

Hemodynamic and blood gas parameters for both the young Long Evans and Eker rats, in both vehicle and PF-4708671-treated groups, fell within the normal ranges for anesthetized rats (refer to Table 1). Mean arterial pressures were significantly higher in Eker rats treated with either vehicle or PF-4708671 compared to the corresponding Long Evans groups. Heart rates did not exhibit significant differences between Long Evans and Eker rats. Treatment with PF-4708671 did not significantly influence arterial blood pressure or heart rate in either group. Arterial blood gases were well-controlled and did not show significant variations across the groups.

**Table 1:** Hemodynamic and blood gas parameters in the Long Evans and Eker rats

	Long Evans (Control)		Eker	
Treatment Group	Vehicle	PF-4708671	Vehicle	PF-4708671
Mean blood pressure (mm Hg)	73 ± 5	62 ± 4	103 ± 10*	98 ± 4*
Heart rate (beats/min)	352 ± 21	343 ± 22	372 ± 12	336 ± 13
Arterial pO_2_ (mm Hg)	112 ± 10	123 ± 8	109 ± 3	114 ± 11
Arterial pCO_2_ (mm Hg)	31 ± 4	30 ± 3	30 ± 3	30 ± 2
Arterial *p*H	7.38 ± 0.03	7.33 ± 0.05	7.38 ± 0.02	7.36 ± 0.02

Values are mean ± SEM (N = 8 per treatment group). *Significantly different from Long Evans values (*p* < 0.05)

**Fig. 1:**
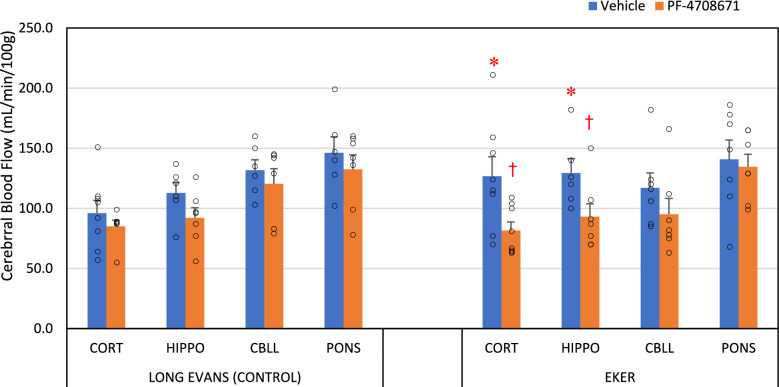
Regional cerebral blood flow in the control (Long Evans rats (LE)) and Eker rats. These rats were vehicle treated (blue bars) or treated with PF-4708671 (red bars). The measured regions were cortex (CORT), hippocampus (HIPPO), cerebellum (CEREB) and pons. In the Long Evans rats, the reduction in cerebral blood flow with PF-4708671 was not statistically significant. The vehicle treated Eker rats had significantly higher cerebral blood flow in the cortex and hippocampus compared to Long Evans rats. Cerebral blood flow was significantly reduced by PF-4708671 in the cortex and hippocampus of the Eker rats (n = 8 per treatment group). Values are mean ± S.E.M. *Indicates a value different from the comparable regions of the Long Evans rats (*p* < 0.05). † indicates a value different from the comparable vehicle treated group (*p* < 0.05)

Under basal conditions, regional cerebral blood flows were significantly elevated in the cortex and hippocampus of Eker rats treated with vehicle compared to the corresponding Long Evans rats (refer to Fig. 1). Specifically, cortical blood flow was approximately 32% higher, and hippocampal flow was around 15% higher in the vehicle-treated Eker rats compared to their Long Evans counterparts. However, no significant differences in blood flow were observed between the cerebellum and pons of the vehicle-treated Eker and Long Evans rats. Following PF-4708671 treatment, a significant reduction in cortical and hippocampal blood flow was observed in the Eker rats, although this decrement was not statistically significant in the cerebellum or pons. Conversely, PF-4708671 administration did not induce statistically significant changes in cerebral blood flow in any examined brain region of the Long Evans rats.

**Fig. 2:**
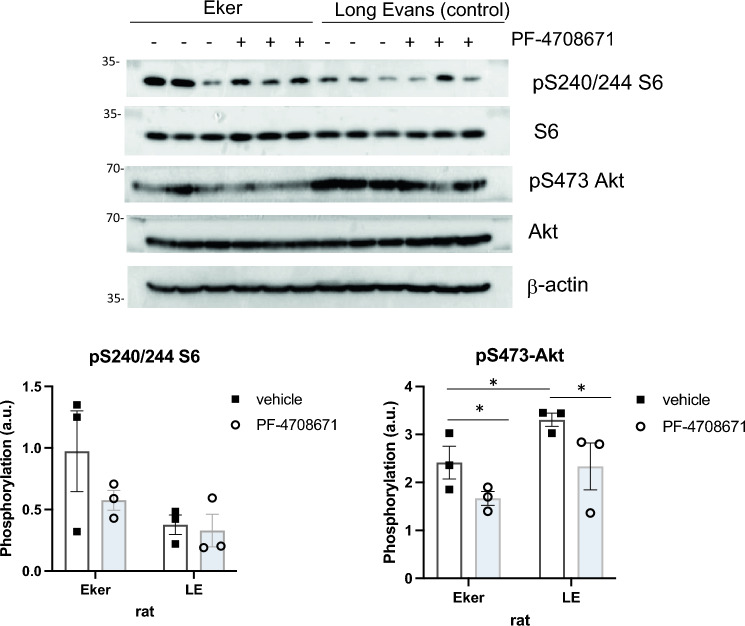
Phosphorylation levels for S6 and Akt are shown for the Control Long Evans and Eker rat brain. Protein expression in the cortices of the Long Evans and Eker rats treated with vehicle or PF-4708671 (S6K1 inhibitor) is shown for three animals for each treatment group. Mean phosphorylation of pS240/244-S6 was higher in the Eker rat compared to the Long Evans in the basal condition. PF-4708671 produced a modest reduction in the Eker rats but not in the Long Evans rats. Results did not reach statistical significance for S6 phosphorylation. Phosphorylation of pS473-Akt was lower and statistically significant in the Eker relative to Long Evans rats. PF-4708671 produced a modest but statistically significant decrease in Akt phosphorylation in both groups. Phosphorylation levels were quantitated and plotted (a.u. arbitrary units) (n = 3 per treatment group). LE: Long Evans. *p < 0.05 Error bars represent S.E.M

To understand the molecular mechanisms underlying the increased cortical blood flow in Eker rats and how PF-4708671 mitigated this abnormality, we analyzed tissues from the brain cortex. We focused on the mTORC1 and mTORC2 effectors, S6 and Akt, respectively. S6 phosphorylation at the S6K1-targeted sites, Ser240/244, tended to be higher in the Eker rats compared to Long Evans (control) rats although this was not statistically significant (refer to Fig. 2). PF-4708671 treatment led to a reduction in S6-S240/244 phosphorylation in the cortex of Eker rats but this did not reach statistical significance as well. Additionally, we investigated Akt phosphorylation at Ser473. This phosphorylation was lower in the vehicle-treated Eker rat brains compared to Long Evans rats. PF-4708671 treatment had a moderately significant negative effect on Akt phosphorylation at Ser473 in the cortex of both Long Evans and Eker rats.

## Discussion

The primary discovery of this study was the reduction of elevated cortical cerebral blood flow in the Eker rat using PF-4708671, a specific inhibitor of S6K1. Interestingly, PF-4708671 did not significantly impact cerebral blood flow in the control Long Evans rats. We found that PF-4708671 diminished S6 and Akt phosphorylation modestly in Eker rats. However, only the latter reached statistical significance. Thus, PF-4708671 could dampen mTOR signaling moderately and this could have played a significant role in reducing the elevated cortical cerebral blood flow in the Eker rat.

In the basal condition of our current study, we observed significant elevations in cortical and hippocampal blood flow in Eker rats compared to young Long Evans controls. These findings align with previous studies that have reported increased cerebral blood flow (CBF), elevated cerebral oxygen consumption, and enhanced oxygen extraction in this Eker rat model (Weiss et al., 2007, 2012). Previously, we reported no significant effects resulting from the stimulation or blockade of AMPA receptors (Weiss et al., 2009, 2012), GABA_A_ receptor blockade (Weiss et al., 2008), or NMDA receptor blockade (Weiss et al., 2007) on the elevated regional cerebral blood flow (rCBF) in Eker rats. In TSC and ASD, an elevated excitation/inhibition balance has been proposed as one of the causes for seizures and autism-like behavior (Bassetti et al., 2021; Curatolo et al., 2022, 2023; Mizuguchi et al., 2021; Rubenstein & Merzenich, 2003). Vigabatrin, an inhibitor of GABA transaminase and a modulator of excitation/inhibition balance, has been employed clinically and in animal models to alleviate seizures and ASD (Bombardieri et al., 2010; Curatolo et al., 2022, 2023; Specchio et al., 2023). Notably, vigabatrin is known to reduce CBF and glucose metabolism in epilepsy patients (Hosking et al., 2003; Spanaki et al., 1999). The reasons for the lack of success in restoring rCBF in our previous studies by modifying agents that influence excitation/inhibition balance in Eker rats remain unclear. However, it might be connected to abnormalities in both the structure and function of receptors, stemming from various factors such as irregular cellular morphology, changes in the number and shape of synapses, remodeling of the extracellular matrix, inflammation, myelin pathology, and network dysfunction caused by dysregulated mTOR signaling (Bassetti et al., 2021; Curatolo et al., 2023; Czapski et al., 2021; Kútna et al., 2020; Pagani et al., 2021; Peters et al., 2019).

Rutten et al. found that among children with TSC, MRI and EEG revealed diminished perfusion in tubers exhibiting EEG slow waves. Furthermore, over time, children with severe developmental delay exhibited decreased brain CBF compared to those with tubers lacking slow waves and experiencing either no or mild developmental delay (Rutten et al., 2023). In our current study, the absence of brain lesions and seizure activity in the Eker rats that were used, led us to interpret the observed increase in rCBF as indicative of potential subtle pathological changes initiated by the hyperactivity of the mTOR pathway. Notably, the susceptibility of Eker rats to brain lesions can be induced by factors such as irradiation and old age, as demonstrated in previous studies (Kútna et al., 2020; Wenzel et al., 2004). These findings underscore the significance of monitoring cerebral blood flow and EEG in individuals with TSC, as they may serve as valuable markers for the prevention and treatment of seizures, developmental delay, and ASD in TSC.

In the present study, the increased regional cerebral blood flow (rCBF) in the cortex and hippocampus of Eker rats was significantly reduced by PF-4708671, which inhibits S6K1, an effector of mTORC1 (Pearce et al., 2010). Rapamycin also notably decreased the elevated rCBF in Eker rats in our prior study (Chi et al., 2015). These findings indicate that the heightened activity of mTORC1 could be responsible for the elevated CBF in Eker rats. One could speculate that the decreased rCBF caused by PF-4708671 might indirectly indicate a reduction in neuronal excitation/inhibition balance. The implications of reduced cerebral blood flow (CBF) or the normalization of elevated CBF in the development of encephalopathy in TSC should be searched. Since one aspect of epilepsy pathophysiology involves increased oxygen consumption due to neuronal excitation to meet oxygen demand, it follows that rCBF should be elevated. If the local oxygen supply/consumption ratio decreases, it could lead to neuronal damage, contributing to epileptic encephalopathy.

While the young Eker rats used in our study exhibit genetic abnormalities similar to human TSC, they do not display tubers in the brain or seizure activity. Further investigation is warranted to understand the reasons for the elevated rCBF in the cortex and hippocampus observed in this study. Rapamycin has shown effectiveness in mitigating and preventing seizures and/or ASD in an animal models of TSC (Ehninger et al., 2008; Koike-Kumagai et al., 2022; Schneider et al., 2017; Tsai et al., 2012). Clinical trials of rapamycin or its related agent everolimus are going on with some promising results in TSC patients (Cavalheiro et al., 2021; Curatolo et al., 2022, 2023; David N. Franz et al., 2021; French et al., 2016; Krueger et al., 2018; Peters et al., 2019) Our current data suggest that PF-4708671 could be efficacious in reducing seizure susceptibility and ASD in TSC during development, as it demonstrates the ability to reduce rCBF similar to rapamycin.

Nevertheless, it is worth noting that rapamycin blocks a large portion of mTOR signaling, potentially causing alterations in cell cycle, protein synthesis, and brain homeostasis resulting numerous side effects (Ganesan et al., 2019). Inhibition of specific downstream targets of mTOR signaling may offer a more selective approach, potentially making it more beneficial for ASD therapy. We used PF-4708671, which is a highly specific inhibitor of S6K1 and of benefit in several neurological disorders, to conduct the current study (Hayashi et al., 2016; Pearce et al., 2010; Srivastava et al., 2016). The decreased cerebral blood flow resulting from the blockade of the S6K1 branch of mTOR signaling could potentially offer therapeutic benefits in the treatment of tuberous sclerosis-ASD in humans (Gkogkas et al., 2013; Kelleher & Bear, 2008; Sharma & Mehan, 2021; Winden et al., 2018). This effect was specific to our tuberous sclerosis-ASD model, as PF-4708671 did not impact cerebral blood flow in the control rats.

We chose to examine whether inhibiting the best-known rapamycin-sensitive mTORC1 target, S6K1, could have a positive effect on restoring normal cerebral blood flow in the Eker rat. S6K1 targets various substrates, including the ribosomal protein S6, that are involved in protein synthesis, metabolism and cell proliferation (Pearce et al., 2010; Tavares et al., 2015). There have been several suggestions that blocking S6K1 may be of benefit in some other models of ASD (Aryal et al., 2021; Bhattacharya et al., 2016). Our results indicate that S6 phosphorylation was increased in two out of the three Eker rats that were examined in comparison to the control Long Evans cohort. One Eker rat seemed to be an outlier and had low basal S6 phosphorylation. An increased S6 phosphorylation would be consistent with enhanced mTORC1/S6K1 signaling that is linked to *Tsc* mutations as in the Eker rat. Additionally, during *Tsc* mutations, a negative feedback loop has been reported to occur such that increased mTORC1/S6K1 downregulates PI3K/mTORC2/Akt signaling (Harrington et al., 2005). Consistent with this notion, we observed a statistically significant decrease in Akt-Ser473 phosphorylation in the vehicle-treated Eker versus Long Evans rats.

Administration of PF-4708671 diminished S6 phosphorylation of two out of the three Eker rats that were examined. However, our results did not reach statistical significance likely due to the one outlier Eker rat. It is also possible that changes in S6 phosphorylation may not be apparent since we are measuring basal levels, i.e., without the presence of activating stimuli. Based on previous findings, an inhibitory effect on S6 phosphorylation seems to be dependent on stimulatory conditions. Whereas PF-4708671 prevented S6 phosphorylation during growth factor stimulation of cells in culture, it had no discernible effect on non-stimulated cells (Pearce et al., 2010). Interestingly, we also found a modest but statistically significant inhibitory effect of the drug treatment on Akt phosphorylation in both the Eker and Long Evans rats. Previous studies demonstrated that PF-4708671 inhibits S6K1 but not the other members of the AGC kinase family which includes Akt. Hence, the effect of PF-4708671 on Akt phosphorylation is likely via an indirect mechanism. Both negative and positive feedback signaling between the two mTOR protein complexes can occur depending on specific cellular conditions to subsequently restore metabolic homeostasis (Szwed et al., 2021). Further studies are needed to define the molecular mechanisms that reduce cerebral blood flow specifically in the Eker rat during S6K1 inhibition.

In conclusion, we found that PF-4708671 reduced the elevated cortical cerebral blood flow in the Eker rat, while PF-4708671 had no significant effect on cerebral blood flow in the control animals. Despite inhibition of S6K1 signaling by PF-4708671, its specific effector that reduces the elevated cortical cerebral blood flow in the Eker rat would need to be further evaluated. Since the Eker rat can serve as a model of tuberous sclerosis-ASD, this suggests that S6K1 inhibition may play an important role in the treatment of tuberous sclerosis-autism spectrum disorders in man. Further work is necessary to study the role of S6K1 in both tuberous sclerosis and ASD.

## Data Availability

Data are available on request.
